# Hybridization of *Saccharomyces cerevisiae* Sourdough Strains with Cryotolerant *Saccharomyces bayanus* NBRC1948 as a Strategy to Increase Diversity of Strains Available for Lager Beer Fermentation

**DOI:** 10.3390/microorganisms9030514

**Published:** 2021-03-02

**Authors:** Martina Catallo, Fabrizio Iattici, Cinzia L. Randazzo, Cinzia Caggia, Kristoffer Krogerus, Frederico Magalhães, Brian Gibson, Lisa Solieri

**Affiliations:** 1 Department of Life Sciences, University of Modena and Reggio Emilia, via Amendola 2, 42122 Reggio Emilia, Italy; 235140@studenti.unimore.it (M.C.); 204845@studenti.unimore.it (F.I.); 2 Department of Agricultural, Food and Environment, University of Catania, via Santa Sofia, 95123 Catania, Italy; cranda@unict.it (C.L.R.); ccaggia@unict.it (C.C.); 3 VTT Technical Research Centre of Finland Ltd., Tietotie 2, P.O. Box 1000, FI-02044 VTT Espoo, Finland; Kristoffer.Krogerus@vtt.fi (K.K.); Frederico.Magalhaes@vtt.fi (F.M.); 4 Chair of Brewing and Beverage Technology, Technical University of Berlin, Seestraße 13, 13353 Berlin, Germany; brian.gibson@tu-berlin.de

**Keywords:** sourdough yeasts, *Saccharomyces bayanus*, outcrossing, heterosis, aroma compounds, brewing

## Abstract

The search for novel brewing strains from non-brewing environments represents an emerging trend to increase genetic and phenotypic diversities in brewing yeast culture collections. Another valuable tool is hybridization, where beneficial traits of individual strains are combined in a single organism. This has been used successfully to create *de novo* hybrids from parental brewing strains by mimicking natural *Saccharomyces* *cerevisiae* ale × *Saccharomyces* *eubayanus* lager yeast hybrids. Here, we integrated both these approaches to create synthetic hybrids for lager fermentation using parental strains from niches other than beer. Using a phenotype-centered strategy, *S. cerevisiae* sourdough strains and the *S. eubayanus* × *Saccharomyces uvarum* strain NBRC1948 (also referred to as *Saccharomyces bayanus*) were chosen for their brewing aptitudes. We demonstrated that, in contrast to *S. cerevisiae* × *S. uvarum* crosses, hybridization yield was positively affected by time of exposure to starvation, but not by staggered mating. In laboratory-scale fermentation trials at 20 °C, one triple *S. cerevisiae* × *S. eubayanus* × *S. uvarum* hybrid showed a heterotic phenotype compared with the parents. In 2 L wort fermentation trials at 12 °C, this hybrid inherited the ability to consume efficiently maltotriose from NBRC1948 and, like the sourdough *S. cerevisiae* parent, produced appreciable levels of the positive aroma compounds 3-methylbutyl acetate (banana/pear), ethyl acetate (general fruit aroma) and ethyl hexanoate (green apple, aniseed, and cherry aroma). Based on these evidences, the phenotype-centered approach appears promising for designing *de novo* lager beer hybrids and may help to diversify aroma profiles in lager beer.

## 1. Introduction

Interspecific hybridization is an evolutionary force that shapes novel phenotypic and genomic profiles and may lead, via adaptive introgression or polyploidization, to the formation of new species [1,2]. In allodiploid hybrids, two divergent sub-genomes integrate and evolve within the same nucleus, with different possible phenotypic outcomes [3]. Genes that have never been tested together by natural selection may generate negative epistatic incompatibility, resulting in phenotypically intermediate hybrids that are suboptimally adapted compared to the parents [4,5]. An alternative outcome of hybridization, known as transgression or heterosis, involves the introgression of selectively favored alleles from one species into another [6]. These superior (over-dominant) combinations of heterozygous loci or the reciprocal complementation of harmful mutations can create hybrids that are able to thrive in new habitats relative to the parents [7].

The *Saccharomyces* genus encompasses many industrially important yeast species, including the model organism *Saccharomyces cerevisiae*. Interspecies sequence divergences range from ~7% between the sister species *Saccharomyces uvarum* and *Saccharomyces eubayanus* [8] to ~25% between *S. cerevisiae* and the members of the *Saccharomyces bayanus* species complex [9,10]. Despite these divergences, all the species tested to date can form F1 hybrids that reproduce asexually by mitosis and are isolated by postzygotic sterility barriers [11]. Natural *S. cerevisiae* × *S.* non-*cerevisiae* hybrids have been frequently isolated from stressful, industry-related niches, such as wine, beer and spirit fermentations, suggesting that hybridization could provide adaptation against stressors and could be successfully exploited in industrial innovation [11,12,13,14,15].

Lager beer, currently the most popular alcoholic beverage worldwide, is produced through wort fermentation at low temperatures (usually between 7 and 15 °C) by *Saccharomyces pastorianus*, an interspecific hybrid between the maltotriose-fermenting yeast *S. cerevisiae* and the cold-tolerant *S. eubayanus* [8,16,17,18,19,20,21]. Lager strains exhibit tolerance to low temperatures and efficient oligosaccharide utilization, two relevant traits in lager brewing environments [20,22,23]. Frohberg-type *S. pastorianus* strains and several Saaz-type *S. pastorianus* strains retain the ability to utilize maltotriose, the second major sugar in wort after maltose. By contrast, this trait can be absent in *S. cerevisiae* ale strains that are responsible for ale brewing [24]. Additionally, Saaz-type strains produce lower concentrations of aroma compounds like ethyl acetate, 3-methylbutanol, and 3-methylbutyl acetate than the more aroma-rich Frohberg yeasts [22].

Even if lager strains are the powerhouse of the modern brewing industry, their phenotypic potential is limited due to their containing genetic material from only two or three individual yeast lineages [17,25,26]. Therefore, the brewing industry looks toward novel brewing starters to meet the consumer demand for product diversification [27]. In response to this demand, several laboratory-made *S. pastorianus*-like hybrids were constructed between *S. cerevisiae* ale strains and *S. eubayanus* to expand the functional repertoire of lager strains [21,28,29]. These synthetic hybrids showed lager brewing performance similar to that of *S. pastorianus* strains, with respect to low-temperature tolerance and maltotriose utilization, but increased aroma diversity in beer compared to beers produced by traditional lager yeasts. However, since its discovery in 2011 in Patagonia [8], *S. eubayanus* is geographically restricted to North America [30], Asia [31], and New Zealand [32] and the low number of available strains hampers the potential of *in vitro* hybridization. Thus, alternative cold-tolerant *Saccharomyces* species, such as *S. uvarum*, *Saccharomyces arboricola* and *Saccharomyces mikatae*, were proposed in hybrid partnership with *S. cerevisiae* [33].

Hybridization can take place only between mating-competent cells. In conventional crossbreeding, sporulation has been utilized to generate mating-competent *MAT*a and *MAT*α haploid cells from non-mater *MAT*a/α diploid cells. In lager and ale strains polyploidy and aneuploidies prevent the progression of meiosis and/or creation of viable spores, limiting the utilization of this technique. Rare mating can bypass the low propensity of brewing yeasts to sporulate as it is based on spontaneous mating caused by loss of heterozygosity at the *MAT* locus [34]. However, interspecies hybridization occurs at a relatively low rate by rare mating and, like mass mating of spores, it requires preliminary time-consuming isolation of auxotrophic parental mutants [29,34,35]. The efficiency of interspecies mating can be improved by genetic modification (GM) techniques, for example, by *HO* deletion followed by interspecies crosses [36,37]. Alternatively, overexpression of the *HO* gene induces illegitimate mating type switching and produces mating-competent *MAT*a/*MAT*a and *MAT*α/*MAT*α diploids at high frequency [38,39]. These strategies produce GM hybrids that are still met with skepticism by consumers, who are reluctant to consume foods made via GM technology.

Another solution to expand the portfolio of available brewing strains is to exploit strains from alternative food-related niches, such as sourdough, Brazilian spirits and sake [40,41,42,43,44,45]. However, these strains typically lack the ability to ferment wort at low temperature and have been proposed for ale brewing and other specialty beer styles [44]. In a previous study, for example, sourdough strains were proven to have good brewing aptitude and to produce “sahti” beer enriched in flavor compounds [45]. Here, we combined bioprospecting for novel yeasts from niches alternative to beer and hybridization and constructed interspecific hybrids between these *S. cerevisiae* sourdough strains and the cold-tolerant *S. bayanus* NBRC1948. This strain was recently demonstrated to be an *S. eubayanus* × *S. uvarum* hybrid with some introgression from *S. cerevisiae* [8,30,46,47]. We evaluated the best conditions to increase hybridization yield using a marker-less spore-to-spore method. We also compared the technological behavior of the obtained triple hybrids at laboratory scale in comparison to *S. cerevisiae* × *S. uvarum* hybrids. Finally, the potential application of the best *S. cerevisiae* × NBRC1948 hybrid candidate was demonstrated in two 2 L-scale fermentation trials.

## 2. Materials and Methods

### 2.1. Strains, Culture Conditions and Chemicals

Strains used in the present work are listed in Table 1. For growth, yeasts were cultured on YPDA medium (1% *w*/*v* yeast extract, 1% *w*/*v* peptone, 2% *w*/*v* dextrose, 2% *w*/*v* agar) for 48 h at 28 °C and then stored at 4 °C for the duration of work. For long-term storage, the strains were maintained at –80 °C in YPD medium (1% *w*/*v* yeast extract, 1% *w*/*v* peptone, 2% *w*/*v* dextrose) supplemented with 25% (*v*/*v*) glycerol as cryopreservation agent. All media and chemicals used in this study were purchased from Sigma Aldrich (St. Louis, MO, USA), except where differently indicated.

Strains used in the present work are listed in Table 1. For growth, yeasts were cultured on YPDA medium (1% *w*/*v* yeast extract, 1% *w*/*v* peptone, 2% *w*/*v* dextrose, 2% *w*/*v* agar) for 48 h at 28 °C and then stored at 4 °C for the duration of work. For long-term storage, the strains were maintained at –80 °C in YPD medium (1% *w*/*v* yeast extract, 1% *w*/*v* peptone, 2% *w*/*v* dextrose) supplemented with 25% (*v*/*v*) glycerol as cryopreservation agent. All media and chemicals used in this study were purchased from Sigma Aldrich (St. Louis, MO, USA), except where differently indicated.

### 2.2. Maltose and Glucose Consumpion Tests

Maltose and glucose fermentation tests were carried out utilizing the procedure described by Kurtzman et al. [50]. Briefly, basal medium (0.45% *w*/*v* yeast extract, 0.75% *w*/*v* peptone and 0.0048% *w*/*v* bromothymol blue as pH indicator) was previously autoclaved, then sugar stock solution was added to a final concentration of 2% (*w*/*v*), and dispensed in test tubes with screw caps and containing inverted Durham tubes. The tubes were inoculated with 10 µL of microbial suspension, kept at 28 °C and monitored for 21 days for the production of gas. Scores were attributed according to Kurtzman et al. [50] as follows: +, strongly positive, insert filled within 7 days; s, slowly positive, insert slowly filled after more than 7 days; −, no gas production.

Growth ability was also evaluated by inoculating each strain at the final concentration of 10^5^ cell/mL in YNB medium (0.67% *w*/*v* Yeast Nitrogen Base with amino acids and ammonium sulfate, BD Difco, Sparks, MD) supplemented with 2% (*w*/*v*) either glucose or maltose as carbon sources. Optical absorbance was measured at 600 nm after 48 h of incubation at 25 °C under shaking conditions (150 rpm) in three independent replicates [40].

### 2.3. Sporulation Efficiency, Spore Viability and Generation of Spore Clones

For sporulation tests, yeasts were sub-cultured in YPDA medium at 28 °C for 24 h, transferred to sporulation medium (ACM; 0.5% *w*/*v* sodium acetate, 2% *w*/*v* agar; pH 6.5) and incubated at 28 °C for a period of 14 days. Asci formation was microscopically checked after 3, 7, and 14 days and scored according to Kurtzman et al. [50].

Sporulation efficiency was determined by resuspending cells/asci mixtures from sporulation medium into physiological water (approximately 1 × 10 μL-loop into 1 mL physiological water). The total number of cells and the number of asci were counted in a Bürker chamber under an optical microscope (Carl Zeiss, Oberkochen, Germany). Sporulation efficiency was calculated as follows:

Sporulation % = [(n° asci)/(n° asci + n° cells)] × 100.(1)

For each strain, at least six tetrad asci were gently digested with 0.2 U of Zymolyase 20 T (AMSBIO, Abingdon, UK) for 20 min at RT and dissected on YPDA plates using a Singer MSM Manual micromanipulator device (Singer Instruments, Somerset, UK), according to [49]. Single spores were incubated at 28 °C for 48 h. Spore viability was calculated as follows:

Spore Viability % = (n° of vital spores/n° of dissected spores) × 100.(2)

The obtained spore clones were streaked on YPDA plates and stored at 4 °C for the duration of work.

### 2.4. Mating Competence Assay

Spore clones were tested for mating competence in mixture cultures with either BY4741 (*MAT*a) or BY4742 (*MAT*α) mating testers on YPDA plates according to Kurtzman et al. [50]. Plates were incubated at 28 °C and conjugative bridges were checked at 8, 24 and 48 h after mixing the cultures.

### 2.5. Construction of Inter-Specific Hybrids

**Figure 1:**
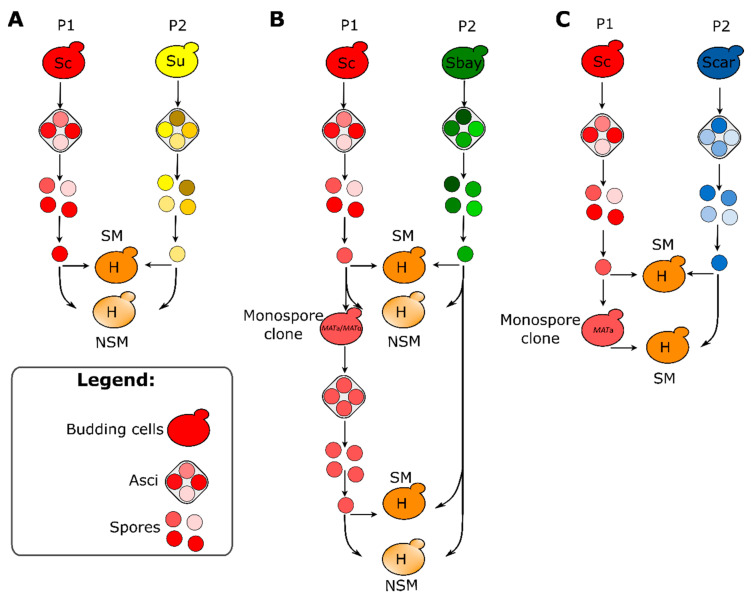
Overview of hybridization scheme used in the present study. Three crosses were attempted: (**A**) *Saccharomyces cerevisiae* × *Saccharomyces uvarum* RC2-10, (**B**) *S. cerevisiae* × *Saccharomyces bayanus* NBRC1948 and (**C**) *S. cerevisiae* × *Saccharomyces cariocanus* CBS8841. *S. cerevisiae* × *S. uvarum* RC2-10 and *S. cerevisiae* × *S. bayanus* NBRC1948 crosses were carried out in staggered mating (SM) and non-staggered mating (NSM), while *S. cerevisiae* × *S. cariocanus* cross in SM only. Mating involved spores from wild-type strains and/or spores from monospore clones in *S. cerevisiae* × Scheme 2. and *S. cerevisiae* × *S. bayanus* NBRC1948 crosses. Mating involved either spores from wild-type strains or mating-competent cells in *S. cerevisiae* × *S. cariocanus* CBS8841 cross. Abbreviations: SM, staggered mating; NSM, non-staggered mating; Sc, *S. cerevisiae*; Su, *S. uvarum*; Sbay, *S. bayanus*; Scar, *S. cariocanus*; P1, parental strain 1; P2, parental strain 2.

Hybrids were generated by the spore-to-spore mating method [49], with the exception of the *S. cerevisiae* × *Saccharomyces cariocanus* cross, where *S. cerevisiae* vegetative haploid monospore clones were used instead of spores. A scheme of hybridization strategy is reported in Figure 1.

In detail, for each mating trial, at least three independent crosses were attempted. *S. cerevisiae* has been reported to have a germination time lower than other *Saccharomyces* non-*cerevisiae* species and this difference negatively impacts interspecies mating [51]. In non-staggered mating (NSM) trials both parental asci were dissected on YPDA mating plates and spores were immediately placed in contact with one another. In staggered mating (SM) trials, *S. cerevisiae* asci were dissected 4 h later than the *Saccharomyces* non-*cerevisiae* asci in order to discern whether hybridization yield increases in SM compared to NSM assays. Mating plates were incubated at 30 °C for 3–4 h and hybrid candidates were streaked at least in duplicate in YPDA medium and then cultured in YPD broth for approximatively 20 generations in order to assure genetic stability of the new hybrids. Finally, hybrids were cryo-preserved at −80 °C in YPD medium supplemented with 25% (*w*/*v*) glycerol.

Hybridization frequency was calculated as follows:

Hybridization frequency (%) = [(n° of positive hybrids scored/n° of total crosses attempted)] × 100.(3)

To test the effect of residence time in starvation conditions on mating propensity, cells were incubated on ACM medium and the plates containing spores were stored at 4 °C for 105 days. Asci age was calculated in days starting from the plating on sporulation medium. Mating propensity was calculated by spore-to-spore mating after 35 and 105 days of residence on ACM, respectively.

### 2.6. Molecular Methods

Yeast DNA was extracted from single colonies with the lithium acetate-SDS method [52]. *MAT* genotyping was carried out on sourdough strains and their monospore clones using primers described by Huxley et al. [53]. Strains BY4742 (*MAT*α) and BY4743 (*MATa*) were used as internal controls. Hybrids were validated by PCR-RFLP analysis of ITS1 spacer with *Hae*III enzyme (Thermo Fisher Scientific, Waltham, MA, USA) [54] and PCR amplifications of *FSY1* and *MEX67* genes using species-specific primers [55]. Genotyping of hybrids after genome stabilization was done by (GTG)_5_ fingerprinting assay according to Dakal et al. [56]. All PCR reactions were carried out in a T100 Thermal Cycler (Bio-Rad, Hercules, CA, USA). All PCR mixtures were performed in 20 μL of final volume containing 1 μL of colony DNA as template, 0.4 µM of each primer, 200 µM each dNTP, and 0.5 U DreamTaq DNA polymerase (Thermo Fisher Scientific, Waltham, MA, USA) according to the manufacturer’s instructions.

### 2.7. Wort Fermentations

Micro-scale trials were carried out according to Catallo et al. [45]. Fermentations were performed in duplicate with 100 mL of 15 °Plato (°P) all-malt wort (96.36 g/L maltose and 40.18 g/L maltotriose) in 250 mL Erlenmeyer flasks, without agitation. Airlocks containing 2 mL of 85% glycerol were used to seal the flasks. Weight loss of the flasks due to CO_2_ release was measured with an analytical balance and used to monitor fermentation progress. A “neutral” fermentation temperature of 20 °C was chosen to support growth of all strains. Final measurements and samples were taken after 14 days. Ethanol concentration and pH values were determined from the centrifuged and degassed fermentation samples using an Anton Paar Density Meter DMA 5000 M with Alcolyzer Beer ME and pH ME modules (Anton Paar GmbH, Graz, Austria). After washing with deionized H_2_O, each yeast pellet was transferred to a pre-weighed porcelain crucible, dried overnight at 105 °C and weighed to determine the dry mass content. Viability percentage was determined in a NucleoCounter^®^ YC-100™ as previously reported [57] and calculated as follows:

% viability = [(total cells − dead cells)/total cells] × 100.(4)

Fermentation curves were modeled based on the weight loss trend over time using the “grofit”-package for R [58]. Maximum rate of fermentation (µ) and maximum fermentation efficiency (total amount of CO_2_ released at the end of the fermentation; A) were determined using the spline-fitting method in “grofit”.

For 2 L-scale fermentations, an inoculation loop was first used to transfer a small amount of yeast to 50 mL YPD medium and propagated under aerobic conditions on a shaker (100 rpm) at 20 °C. After propagation overnight, the cells were washed in sterile Milli-Q-filtered water and re-suspended to achieve a 20% slurry (200 g fresh yeast/L). This slurry was used to inoculate 1 L of all-malt 15 °P wort in a 2 L Erlenmeyer flask at a pitching rate of 1 g/L. After a two-day incubation period at 20 °C and shaking (100 rpm), the flask contents were centrifuged and yeast was again washed and re-suspended to achieve a 20% slurry. In this case the fermented wort, rather than water, was used for resuspension of cells. This slurry was used to inoculate 1.5 L of 15 °P wort within 2 L-scale sterile stainless steel cylindroconical vessels at a pitching rate of 1 g/L. Fermentations were conducted at 12 °C, as similarly low temperatures are typical for the production of lager-style beer, and low-temperature tolerance is an essential criterion for the selection of prospective lager brewing yeasts. Samples were taken aseptically throughout the fermentation period. Samples for yeast-derived flavor compound determinations were drawn from the beer when fermentations were ended and immediately frozen until the analysis.

### 2.8. Chemical Analysis

Yeast-derived flavor compounds were determined by headspace gas chromatography with flame ionization detection (HS-GC-FID). Briefly, samples of 4 mL were filtered (0.45 µm), incubated at 60 °C for 30 min, and then 1 mL of gas phase was injected (split mode; 225 °C; split flow of 30 mL/min) into a gas chromatograph equipped with an FID detector and headspace autosampler (Agilent 7890 Series; Palo Alto, CA, USA). Analytes were separated on a HP-5 capillary column (50 m × 320 µm × 1.05 µm column, Agilent, Palo Alto, CA USA). The carrier gas was helium (constant flow of 1.4 mL/min). The temperature program was 50 °C for 3 min, 10 °C/min to 100 °C, 5 °C/min to 140 °C, 15 °C/min to 260 °C and then isothermal for 1 min. Compounds were identified by comparison with authentic standards and were quantified using standard curves. 1-Butanol was used as internal standard (246 mg/L).

For sugar analysis, the samples collected at the end of the fermentation were centrifuged and the supernatants were filtered (0.45 µm) prior to storing in a freezer (–23 °C). Sugar content of wort was analyzed by HPLC. A Waters 2695 Separation Module and Waters System Interphase Module liquid chromatograph coupled with a Waters 2414 differential refractometer (Waters Co., Milford, MA, USA) was used. An Aminex HPX-87H Organic Acid Analysis Column (300 × 7.8 mm; Bio-Rad, Hercules, CA, USA) was equilibrated with 5 mM H_2_SO_4_ (Titrisol, Merck, Germany) in water at 55 °C, and samples were eluted with 5 mM H_2_SO_4_ in water at a 0.3 mL/min flow rate.

### 2.9. Statistical Analysis

Data on maltose and glucose consumption and on 2 L wort fermentation were statistically analyzed with two-way ANOVA (*p* < 0.05) followed by a Tukey’s multiple comparisons test. Statistical analysis was performed on kinetics parameters and aroma concentrations with a one-way ANOVA and Tukey’s test. All analyses were performed using GraphPad Prism software (GraphPad Software, Inc, San Diego, CA, USA). *p* values < 0.05 were considered as statistically significant.

## 3. Results

### 3.1. Maltose Fermentation Screening

Maltose is one of the main fermentable sugars in sourdough because it is constantly generated by the degrading activity of amylase on starch [59]. Maltose represents 60% of carbohydrates in wort, a complex substrate also containing 10% of glucose and 25% of maltotriose as fermentable sugars [60]. Sourdough strains were proven to exhibit a good brewing aptitude [45] and could be promising parental candidates for brewing hybrid construction with *Saccharomyces* non-*cerevisiae* cryotolerant species. Fermentation tests showed that all *S. cerevisiae* strains, *S. uvarum* RC2-10, *S. bayanus* NBRC1948 ferment maltose, but not *S. cariocanus* CBS8841 (Table 2).

To assess inter-strain differences in growth on maltose, biomass was also tested as absorbance after 48 h of fermentation in maltose and glucose-containing media. *S. cerevisiae* Y19, Y21, Y23 and Y26 were the best maltose-fermenting strains, while Y17 exhibited a slow-growing phenotype both in glucose and maltose-supplemented media. Strains Y19 and Y23 did not show any significant diversity in maltose and glucose consumption (*p* < 0.05) (Table 2).

### 3.2. Sporulation Efficiency and Spore Viability

In order to generate artificial hybrids by spore-to-spore mating, parental strains should sporulate and produce viable spores. Table 3 shows that the majority of strains sporulated after 3 days of incubation with a sporulation efficiency ranging from 17.2 to 35.9%. *S. cerevisiae* Y17 was the best sporulating strain (35.9%), while three strains, including *S. uvarum* RC2-10, and *S. cerevisiae* Y18 and Y26, showed an efficiency lower than 25%. Despite Y17 having the highest sporulation efficiency, it showed the lowest spore viability (29.2%), while other yeasts had spore viability values ranging from 45.8% for strains Y18 and Y19 to 66.7% for strain Y23. Overall, *S. cerevisiae* Y15, Y19, Y21 and Y23 exhibited the best phenotype in terms of both sporulation efficiency and spore viability. Three of them (Y19, Y21 and Y23) were the most suitable to grow on maltose as carbon source.

### 3.3. MAT Genotyping of Strains and Their Meiotic Segregants

Yeasts with *MAT*a/*MAT*α genotype are presumed to be non-haploid strains [61]. *S. cerevisiae* sourdough strains had a *MAT*a/*MAT*α genotype (Table 3): this genotype could be congruent with a diploid status, but aneuploidy and/or polyploidy (such as *MAT*a/*MAT*a/*MAT*a/*MAT*α, *MAT*α/*MAT*α/*MAT*α/*MAT*a and *MAT*α/*MAT*α/*MAT*a/*MAT*a) cannot be excluded.

**Figure 2:**
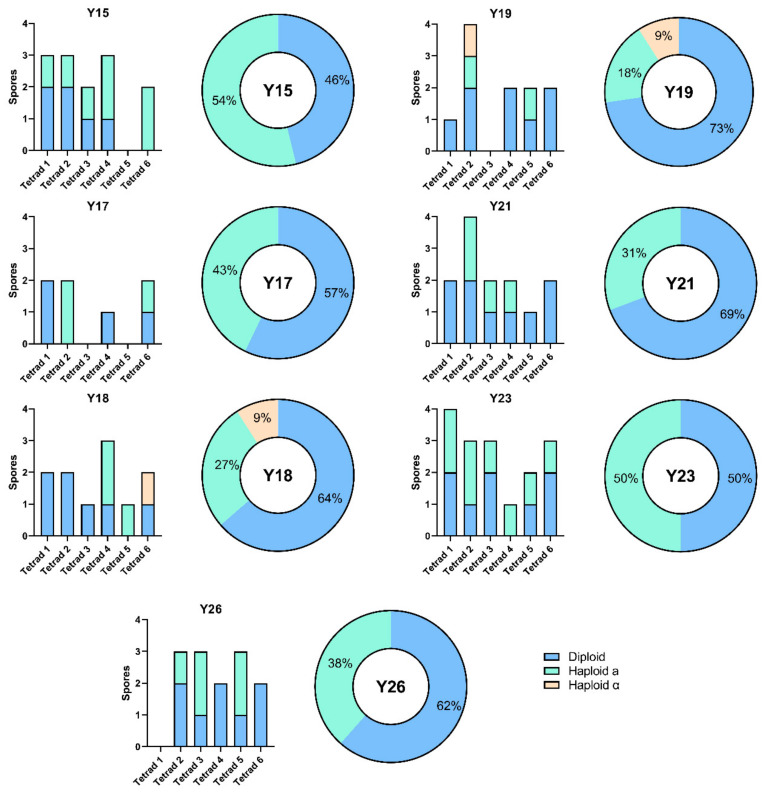
Mating types of meiotic segregants from *S. cerevisiae* sourdough strains. For every strain, column graph (**left**) shows *MAT* loci segregation in single meiotic events, while pie chart (**right**) Table.

Homothallism/heterothallism are critical factors that affect mating efficiency and hybridization yield [62]. Homothallic *HO*/*HO* diploid strains would result in tetrads with only diploid *MAT*a/*MAT*α spores, while heterothallic *ho*/*ho* strains would be associated with *MAT*a and *MATα* progenies in a maximal ratio of 2:2, excluding death events. Conversely, heterozygous *HO*/*ho* strains can generate spores with either diploid or haploid genomes in a typical ratio of 2:2. To unravel the ability of sourdough strains to switch mating type, *MAT* multiplex PCR was also carried out on their meiotic segregants. All strains yielded the *MAT*a/*MAT*α segregant progenies with a frequency ranging from 46% to 73% (Figure 2). We also found *MAT*a segregant clones (Figure 2) unable to sporulate and able to mate with mating tester BY4742 (*MAT*α) (data not shown). All these results support that the *MAT*a meiotic segregants are mating-competent and therefore suitable for outcrossing. Interestingly, *MAT*α clones were rarely dissected, with the exception of two tetrads from strains Y18 and Y19 (Figure 2). As BY4742 (*MAT*α) was used as a positive control in PCR amplification of the *MAT*α locus, we supposed that the majority of *MAT*α haploid cells might be non-viable. Analysis of progeny distributions within each tetrad showed that diploid *MAT*a/*MAT*α occurred no more than twice for tetrad (Figure 2). The coexistence of *MAT*a/*MAT*α and *MAT*a spores in the same tetrad is congruent with a *HO*/*ho* genotype for the tested strains, suggesting that they are suitable for spore-to-spore mating.

### 3.4. Hybrid Construction

Sourdough strains Y15, Y19, Y21 and Y23 were chosen for hybrid construction on the basis of maltose consumption, sporulation efficiency and spore viability. Strain NBRC1948 (referred to as Sbay for convenience) was chosen for its peculiar genetic content, as being an *S. eubayanus* × *S. uvarum* hybrid with introgressed segments from *S. cerevisiae* [30]. *S. uvarum* RC2-10 (referred to as Su for convenience) and *S. cariocanus* CBS8841 (referred to as Scar for convenience) were chosen as alternative cryotolerant parents for comparative purposes.

Out of 190 attempted crosses, a total of 46 hybrids were generated and validated by molecular ITS1 PCR-RFLP assay and species-specific PCR (Supplementary Figure S1). Hybrid yield was higher than 22% for most of the cases (Table 4). Out of 46 novel hybrids, three were between *S. cerevisiae* and *S. cariocanus* (Sc × Scar), 11 between *S. cerevisiae* and *S. uvarum* (Sc × Su), and 32 between *S. cerevisiae* and *S. bayanus* (Sc × Sbay). All three Sc × Scar hybrids, as well as three Sc × Su and 11 Sc × Sbay randomly selected hybrids were tested for sporulation. As opposed to Sc × Su hybrids, all the Sc × Sbay and Sc × Scar hybrids were able to sporulate (Supplementary Table S1).

Differences in germination time are some of the main prezygotic barriers among *Saccharomyces* species [63], so we performed pilot mating experiments under NSM and SM conditions. In the case of the *S. cerevisiae* × *S. uvarum* cross, we first attempted to gain hybrids by SM, which was previously reported to enhance hybridization yield [64]. Under SM, hybridization frequency was 27% for Y23 × RC2-10 crosses, whereas no hybrids were obtained when spores of *S. cerevisiae* Y15 were mated with spores of *S. uvarum* RC2-10 (Table 4). When the same strain pairs were tested under NSM conditions, hybrid frequency ranged between 20 and 31%, suggesting that differences in germination time were not relevant for these parental strains. In mating trials between *S. cerevisiae* and *S. bayanus*, SM did not enhance hybridization frequency compared with the NSM condition (Table 4).

*S. cariocanus* represents a particular *S. paradoxus* strain harboring four chromosomal translocations and which, consequently, is not interfertile with the *S. paradoxus* tester strain [18]. In *S. cerevisiae* × *S. cariocanus* crosses, we considered two individual interspecific pairings under SM, which was previously demonstrated to increase hybridization rates in *S. cerevisiae* × *S. paradoxus* mating trials [51]. Either spores from strain Y19 (*MAT*a/*MAT*α) or vegetative cells of the haploid monospore clone Y19.5A (*MAT*a) were mated with spores from *S. cariocanus* CBS8841. By using 4 h of staggering time in both crosses, we obtained hybrid yields of 22% and 9% for Y19 × CBS8841 (spore-to-spore mating) and Y19.5A × CBS8841 (cell-to-spore mating), respectively.

During these pilot experiments, we also observed that asci maintained on ACM plates for long times (approximately 105 days) resulted in spores particularly prone to mate. To corroborate this observation, we performed Sc × Su and Sc × Sbay mating trials with both spores on ACM for 35 days and spores on ACM for 105 days. As shown in Table 5, old spores resulted in hybridization frequency higher than young spores, suggesting that spore age affects hybridization yield more than SM conditions.

### 3.5. Laboratory-Scale Wort Fermentation

**Figure 3:**
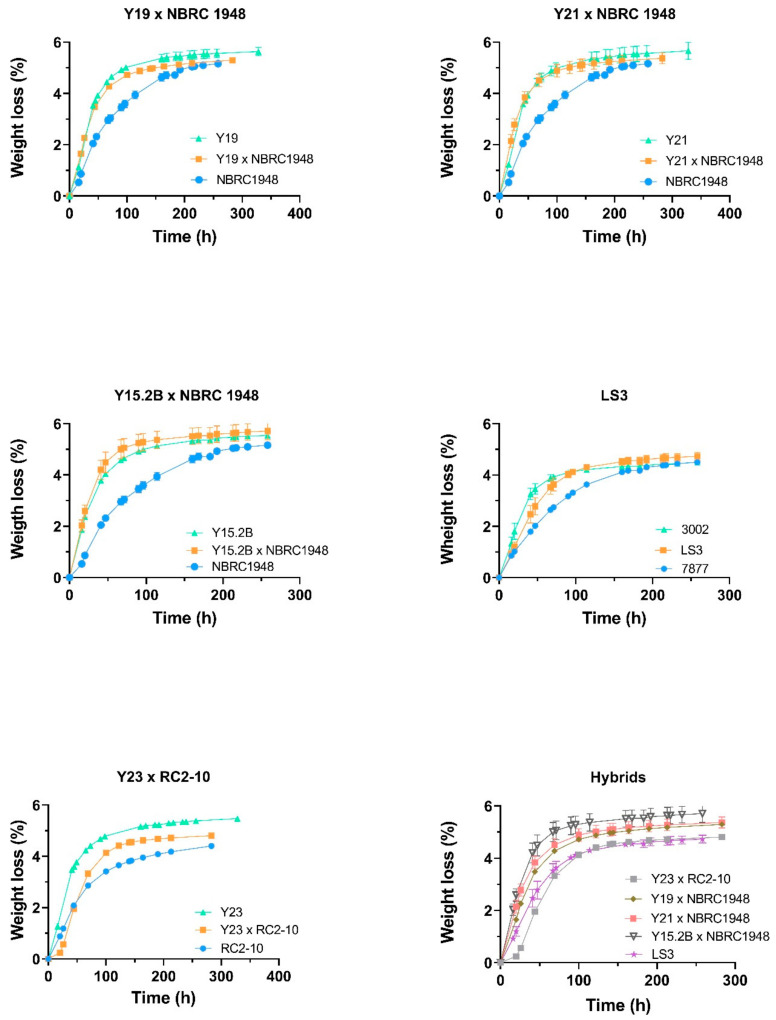
Growth curve plots of hybrids and their parents in wort fermentation assays at laboratory scale (15 °P; 20 °C). Growth kinetics were measured as weight loss percentage over time. Values are mean of three replicates. Bars represent standard deviation.

Novel Sc × Sbay (Y15.2B × NBRC1948, Y19 × NBRC1948 and Y21 × NBRC 1948) and Sc × Su (Y23 × RC2-10) hybrids were selected for laboratory-scale wort fermentation together with their respective parental strains. One synthetic Sc × Su hybrid constructed for wine fermentation, namely LS3, was added for comparative purposes. In Figure 3 and Supplementary Figure S2, fermentation profiles show that Sc × Sbay hybrids retained the fermentation performance of the best Sc parental strains, while Sc × Su hybrids exhibited an intermediate kinetic curve compared with the parents.

**Figure 4:**
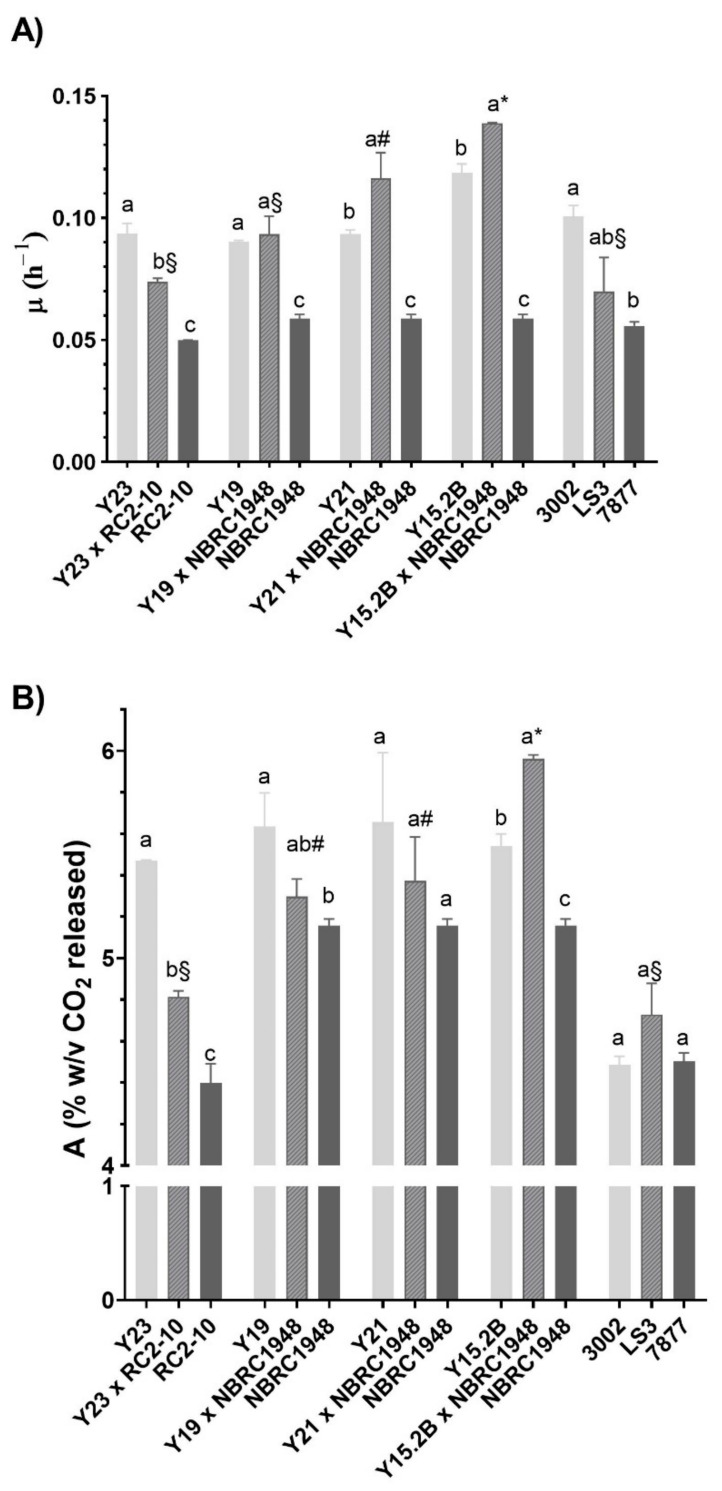
Values of maximum fermentation rate μ (h^−1^) (**A**) and maximum fermentation level A (% *w*/*v* CO_2_) (**B**) in laboratory-scale wort fermentation (15 °P, 20 °C). *S. cerevisiae*, hybrids and *S. bayanus*/*S. uvarum* are represented as solid light gray, hatched gray and solid gray columns, respectively. Different letters indicate significant differences (*p* < 0.05) within each triad of hybrid and parents based on one-way ANOVA; different symbols (§, # and *) indicate significant differences (*p* < 0.05) among hybrids Y23 × RC2-10, Y19 × NBRC1948 and Y15.2B × NBRC1948 based on one-way ANOVA. Superscript letters were attributed as follows: the highest value was marked as “a,” the next value that is significantly different can be “b,” and so on.

Growth curve analysis revealed that Sc × Sbay hybrid Y15.2B × NBRC1948 exhibited higher maximum fermentation rate (μ) and maximum fermentation level (A) (*p* < 0.05) compared with the tested strains. This hybrid fermented faster than other hybrids and slightly faster compared with the Sc parental strain Y15.2B (Figure 4).

Ethanol production ranged from 5.8% to 7.3% *v*/*v* after 12 days of fermentation at 20 °C (Supplementary Table S2). Sc × Sbay hybrids outperformed the Sc × Su hybrid in ethanol yield (*p* < 0.05). In contrast to previous studies involving ale strains crossed with *S. eubayanus* [28,29,65], both Sc × Su and Sc × Sbay hybrids were intermediate in alcohol production compared with the parental strains. Sourdough strains and their hybrids with NBRC1948 yielded ethanol at more than 82% of the theoretical value. Su strains were significantly lower in viability than Sbay NBRC1948 (*p* < 0.05).

### 3.6. Two L-Scale Wort Fermentation

**Figure 5:**
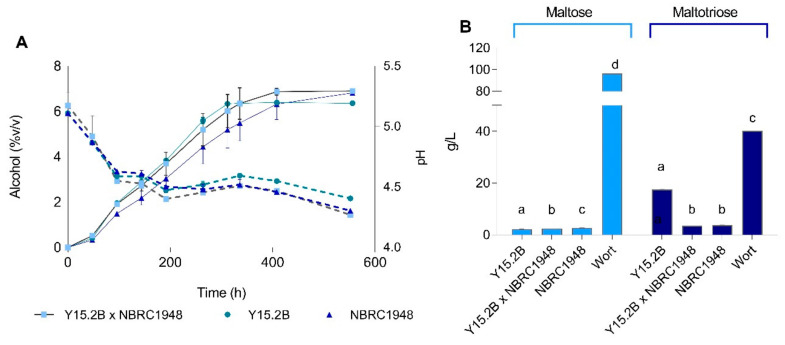
Wort fermentation at 2 L-scale (15 °P, 12 °C). Panel A exhibits trends of alcohol content (% *v*/*v*) (solid line) and pH values (dotted line) over time, while panel B reports residual sugars (maltose and maltotriose) at the end of fermentation. Values are means of two independent replicates; bars represent error range. Different letters indicate significant differences (*p* < 0.05) based on two-way ANOVA.

Based on the laboratory-scale fermentation results, we selected Sc × Sbay hybrid Y15.2B × NBRC1948 for 2 L-scale wort fermentation at 12 °C. Under these conditions, the hybrid exhibited a transgressive phenotype relative to its parents; it was able to ferment efficiently the wort sugars and also to tolerate low temperatures (Figure 5), a trait that was not detectable from the previous small-scale fermentations that were carried out at the higher temperature of 20 °C.

The *S. cerevisiae* parent performed relatively well in the first 10 days of fermentation, but the alcohol yield was limited in the latter stage of the fermentation, apparently due to an inability to utilize efficiently the available maltotriose, which was present in the final beer at approx. 20 g/L (Figure 5). In contrast, the *S. bayanus* parent fermented more slowly, but was more efficient in terms of overall alcohol yield and sugar consumption, with residual sugar concentrations being similar to those of the hybrid strain.

**Figure 6:**
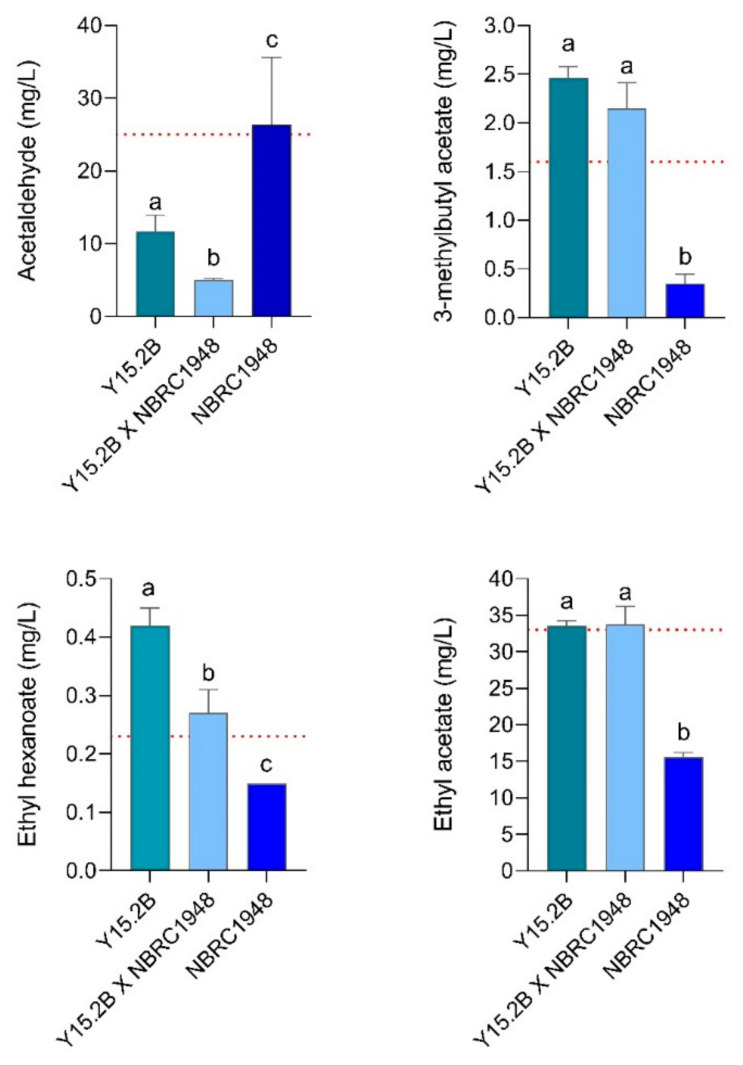
Concentrations of flavor-active compounds (mg/L) after 2 L wort fermentation (15 °P, 12 °C). Values are means of two independent replicates; bars represent error range. Different superscript letters indicate significant differences (*p* <  0.05) as determined by one-way ANOVA and Tukey’s test. For each compound, the relative perception threshold is reported as dotted red line [66].

The benefit of hybridization was seen not only in terms of fermentation performance, but also in beer quality. Four flavor-active aroma volatiles showed positive differences in concentration (Figure 6). Acetaldehyde, which imparts an unpleasant chemical taste to beer and is particularly noticeable in lager beers, was at the flavor threshold in the *S. bayanus* beer. Low levels were observed in the *S. cerevisiae* beers, as well as those beers created with the hybrid strain. 3-methylbutyl acetate, which imparts a banana or pear flavor to beer and is one of the most desirable volatile compounds, was present at concentrations above the flavor threshold in the *S. cerevisiae* and hybrid strain beers. The *S. bayanus* beer concentrations were lower than the typical flavor threshold values and are not expected to contribute in any positive way to the overall flavor profile. A similar result was found for ethyl acetate (general fruit aroma) and ethyl hexanoate (green apple, aniseed, and cherry aroma) (Figure 6).

## 4. Discussion

Bioprospecting efforts in brewing seek to utilize yeasts from environments other than the brewery in order to augment and/or diversify flavor properties of the final product [44]. Sourdough is a bio-reservoir of particular interest due to the occurrence of maltose-positive *S. cerevisiae* strains with QPS/GRAS status [27,67] and potentially well-accepted by consumers for their provenance from artisanal food systems [68]. The search for new *S. cerevisiae* sourdough strains as wild stocks could have great potential for wheat and other specialty beers [40,45]. Our sourdough strains are well-suited both for fermenting maltose and for producing flavorful molecules like ethyl- and acetate-esters [45]. However, they do not display the complete pattern of industrial adaptive signatures specific for lager brewing, making the exploitation of genetic improvement techniques mandatory. The aim of this work was to demonstrate the suitability of a phenotype-centered strategy based on outcrossing of selected strains derived from non-brewing environments for increasing genetic and phenotypic diversity of starter cultures for lager-type fermentations.

Crossbreeding between maltotriose-fermenting *S. cerevisiae* ale yeasts and cryotolerant *S.* non-*cerevisiae* species is effective for creating novel GM-free synthetic hybrids based on mating between cells with opposite mating type by using marker-assisted approaches of spore-to-spore mating, mass mating and rare mating [21,28,29,33,65]. As our sourdough strains are able to sporulate and produce viable spores, they were mated with the cryotolerant *S. bayanus* strain NBRC1948 to generate synthetic hybrids via a marker-less spore-to-spore mating approach. Generally, mass mating is fast procedure that is preferred over the spore-to-spore method and, recently, novel methods such as fluorescence-activated cell sorting (FACS) [69] were proposed for increasing and assisting marker-less hybrid recovery after rare mating. Here we demonstrated that spore-to-spore mating can produce hybrids without the requirement of any selectable phenotypes for the parental strains and at relatively high frequency if the conditions of mating have been optimized. For instance, differences in germination time may contribute to reproductive isolation between species [51,63]. In contrast to what was previously found in outcrossing between *S. cerevisiae* and the phylogenetically distant species *S. uvarum*, isolated spores of *S. cerevisiae* sourdough strains and *S. bayanus* NBRC1948 appeared to germinate almost synchronously and did not require any staggered mating. This may depend on the peculiar genetic make-up of the selected cryotolerant parent NBRC1948, which contains several introgressed *S. cerevisiae* genomic segments [46]. We also found that a prolonged exposure to starvation positively affects mating propensity of isolated spores, enhancing the recovery of Sc × Sbay hybrids. Furthermore, heterothallic parental strains or wild strains with at least one inactive copy of the *HO* gene should be preferred over homothallic yeasts for breeding as isolated spores harboring the inactive *ho* allele are forced to restore diploidy by outcrossing. Here, we found that all sourdough strains gave a pattern of meiotic segregants compatible with a *HO*/*ho* genotype. Despite homothallism representing the most common life cycle in *S. cerevisiae* [60,70], heterothallic strains are frequently found in nature [71,72], while heterozygous *HO*/*ho* strains were also isolated in industrial environments for Brazilian spirit production [73]. The extent of loss of function in *HO* genes from natural sourdough strains deserves further investigations in future. Like in other baker’s yeasts [74], the *MAT*a idiomorph prevails in meiotic segregants, suggesting two possible explanations. *MAT*a monospore clones could be more viable than *MAT*α ones or, alternatively, sourdough strains could have a *MAT*a/*MAT*a/*MAT*a/*MAT*α genotype. Overall, these results highlight the propensity of sourdough strains for outcrossing and provide some guidelines for optimizing successful outcrosses in spore-to-spore mating experiments without any GM technology utilized.

Evaluation of the hybrids under lager brewing conditions clearly showed the potential of the *S. cerevisiae* × *S. bayanus* hybrid combination for industrial application. With one particular parental combination, the hybrid benefited from increased maltotriose utilization and higher concentration of esters in the resultant beers. While yeast hybrids occur in both natural and industrial environments [14,15,75,76,77], they appear to be more common (or more persistent) in the latter. This suggests that the hybrid state confers a distinct advantage in such environments. Recent work involving artificially created hybrids has confirmed this suggestion, with newly created hybrids showing promise for application in a number of fermentation environments including beer, cider, and wine production [24,29,35,78,79,80]. The *de novo* creation of lager yeast hybrids has been of particular interest due to the commercial importance of the *S. pastorianus* lager yeast and the low level of diversity within this group. Recreation of the *S. cerevisiae* × *S. eubayanus* hybrid has shown clearly that the success of *S. pastorianus* under low-temperature wort brewing conditions was due to two-parent transgression. The hybrid benefited from the superior ability of *S. cerevisiae* to utilize wort sugars (particularly maltotriose), and the psychrotolerance of *S. eubayanus*. Hybrids therefore had a competitive advantage over both ale yeasts and wild *Saccharomyces* yeasts when fermenting wort at low temperature [28]. The advantage is, however, not necessarily parent-species specific; any combination of species that introduces the required phenotypes should be effective. Nikulin et al. [33], for example, created yeasts with a lager yeast phenotype by combining *S. cerevisiae* with yeasts other than *S. eubayanus*. *Saccharomyces mikatae*, for example, was equally effective at transferring the cold-tolerant phenotype to the hybrid. There is therefore scope for creating yeasts with industry-relevant phenotypes without recapitulating the original species combination [81]. The example in the present study is the first to show that a yeast with a phenotype suitable for brewing can be created even when neither parent has been used as production strain for brewing. This phenotype-centered approach to parent selection increases the genetic diversity that may be tapped for hybrid design.

Previous attempts to create lager hybrids have utilized *S. cerevisiae* as the maltose-, and maltotriose-positive partner [28]. Of note here was the finding that the *S. bayanus* parent appeared to be more adept at wort sugar utilization than the *S. cerevisiae* parent. This, at first, may seem counterintuitive, given that maltotriose utilization is not typical for either of the species (*S. eubayanus* or *S. uvarum*) included in the species complex [33]. However, the *S. bayanus* strain utilized in this study (NBRC1948) is known to possess a considerable amount of *S. cerevisiae-*introgressed DNA and, in particular, a large (70 kb) region containing the maltose transporter gene *MAL31*, and the maltose and maltotriose transporter gene (*MTY1/MTT1*). These genes and a number of other *S. cerevisiae*-derived genes related to osmotic stress resistance, anaerobic growth, and sucrose utilization may have been key to the survival of this strain as a contaminant in a brewery environment (the original source of the strain) [40]. This same introgression is feasibly responsible for the improved brewing efficiency of the hybrid created in this study. It is perhaps significant here that the Mtt1 transporter is known to have a high affinity for maltotriose relative to other transporters [82], and this may explain the greater ability to consume the trisaccharide during fermentation. It may be hypothesized that the sourdough *S. cerevisiae* parent possesses a lower affinity transporter such as Malx1. It has also been found that the Mtt1 transporter performs well at low temperature relative to other yeast maltotriose transporters [83].

In conclusion, this study proves that synthetic triple hybrids involving phenotype-based selection of non-brewing parents could be useful for diversification of lager yeast stock cultures. Outcrossing experiments defined environmental conditions that optimize hybrid recovery without any utilization of auxotrophic marker and/or GM techniques. In being an *S. eubayanus* x *S. uvarum* hybrid, strain NBRC1948 exhibits cold-tolerance, but is also able to ferment maltotriose better than *S. eubayanus* due to the presence of *MTY1*/*MTT1*, a gene encoding the cold-tolerant maltotriose transporter described in *S. pastorianus* [82,84]. *S. cerevisiae* sourdough strains have good fermentative aptitude in wort and positively affect aroma profile, a phenotype that is positively inherited by hybrids. Phenotype-driven selection of parents followed by outcrossing is a promising biotechnological solution that combines different benefit phenotypes into a single brewing culture.

## Figures and Tables

**Table 1:** Strains used in this work.

Strains	Code	Origin/Characteristics
*S. cerevisiae*	Y15, Y17, Y18, Y19	Sourdough of Maiorca flour (Maletto, CT)
	Y21	Sourdough of Maiorca flour (Castellammare, TP)
	Y23	Sourdough of Maiorca flour (Catania, CT)
	Y26	Sourdough of Maiorca flour (Balestrate, PA)
	BY4741	Euroscarf/*MAT*a, *his3Δ1*, *leu2Δ0*, *met15Δ0*, *ura3Δ0*
	BY4742	Euroscarf/*MAT*α, *his3∆1*, *leu2∆0*, *lys2∆0*, *ura3∆0*
	BY4743	Euroscarf/ *MATa/MAT*α, *his3∆1/ his3∆1*, *leu2∆0/ leu2∆0*, *lys2∆0/ lys2∆0*, *ura3∆0/ ura3∆0*
	3002	Wine [48]
*S. pastorianus*	Fermolager W	Lager Frohberg yeast (AEB spa, Brescia)
*S. uvarum*	RC2-10	Grape fermenting yeast (Alsace); kindly provided by Philippe Marullo
	7877	wine; DIROVAL ^1^ collection
*S. cerevisiae* × *S. uvarum* hybrid	LS3	[49]
*S. bayanus*	NBRC1948	-
*S. cariocanus*	CBS8841	*Drosophila* sp. (Rio de Janeiro, Brazil)

^1^ DIPROVAL, Dipartimento di Protezione e Valorizzazione Agroalimentare, University of Bologna.

**Table 2:** Maltose consumption tests. The results of fermentation tests are scored according to Kurtzman et al. [50] as follows: +, strongly positive; s, slowly positive; -, no gas production. Optical density at 600 nm (OD_600 nm_) was evaluated after 48 h of fermentation in medium containing either glucose or maltose. Different superscript letters indicate significant differences (*p* < 0.05) in the same column, while different symbols (* and §) indicate significant differences in the same raw, as determined by ANOVA followed by Tukey HSD test. Superscript letters were attributed as follows: the highest value was marked as “a,” the next value that is significantly different can be “b,” and so on. Abbreviation: nd, not determined.

Species	Strain	Fermentation Assay	Optical Density (OD_600 nm_)	
			Glucose (2% *w*/*v*)	Maltose (2% *v*/*w*)
*S. cerevisiae*	Y15	s	1.402 ^b,^* ± 0.031	1.098 ^b,§^ ± 0.02
	Y17	s	1.145 ^c,^* ± 0.016	0.677 ^c,§^ ± 0.06
	Y18	s	1.438 ^b,^* ± 0.042	1.029 ^b,§^ ± 0.093
	Y19	+	1.428 ^b,^* ± 0.016	1.257 ^a,b,^* ± 0.046
	Y21	s	1.970 ^a,^* ± 0.203	1.412 ^a,§^ ± 0.05
	Y23	s	1.551 ^b,^* ± 0.228	1.495 ^a,^* ± 0.119
	Y26	+	1.687 ^a,^* ± 0.12	1.252 ^a,b,§^ ± 0.101
*S. uvarum*	RC2-10	s	nd	nd
*S. bayanus*	NBRC1948	s	nd	nd
*S. cariocanus*	CBS8841	-	nd	nd

**Table 3:** Sporulation efficiency, spore viability and *MAT* genotype. Strains sporulating after 3 and 7 days were scored as + and w (weak), respectively. Sporulation efficiency was determined as the mean of two independent replicates, while viability was calculated considering the dissection of six tetrads for each strain. All values of sporulation efficiency were statistically different based on one-way ANOVA. Abbreviation: nd, not determined.

Species	Strain Code	Sporulation	Sporulation Efficiency (%)	Viability (%)	*MAT* Genotype
*S. cerevisiae*	Y15	+	32.9 ± 0.07	47.2	*MAT*a/*MAT*α
	Y17	+	35.9 ± 0.04	29.2	*MAT*a/*MAT*α *MAT*a/*MAT*α
	Y18	+	19.0 ± 0.04	45.8	*MAT*a/*MAT*α
	Y19	+	35.4 ± 0.00	45.8	*MAT*a/*MAT*α
	Y21	+	34.5 ± 0.04	54.2	*MAT*a/*MAT*α
	Y23	+	32.1 ± 0.04	66.7	*MAT*a/*MAT*α
	Y26	w	17.2 ± 0.01	54.2	*MAT*a/*MAT*α
*S. uvarum*	RC2-10	w	20.4 ± 0.01	nd	nd
*S. bayanus*	NBRC1948	w	30.9 ± 0.02	nd	nd
*S. cariocanus*	CBS8841	+	nd	nd	nd

**Table 4:** Hybridization frequency (% values) in staggered (SM) and non-staggered (NSM) mating trials. Abbreviations: SM, staggered mating; NSM, non-staggered mating; Sc, *S. cerevisiae*; Su, *S. uvarum*; Sbay, *S. bayanus*; Scar, *S. cariocanus*; nd, not determined.

Parental Strains	Type of Mating	Hybridization Frequency (%)	
		SM	NSM
Y15 × RC2-10	Sc × Su direct spore-to-spore	0	23
Y23 × RC2-10	Sc × Su direct spore-to-spore	27	31
Y15.2B × NBRC1948	Sc segregant spore-to-Sbay spore	10	41
Y19 × NBRC1948	Sc × Sbay direct spore-to-spore	31	42
Y21 × NBRC1948	Sc × Sbay direct spore-to-spore	13	33
Y19 × CBS8841	Sc × Scar direct spore-to-spore	22	nd
Y19.5A × CBS8841	Sc cell-to-Scar spore	9	nd

**Table 5:** Effect of spore residence in ACM on hybridization frequency. Abbreviations: SM, staggered mating; NSM, non-staggered mating; Sc, *S. cerevisiae*; Su, *S. uvarum*; Sbay, *S. bayanus.*

Parental Strains	Type of Mating	Hybridization Frequency (%)			
		35 Days Age		105 Days Age	
		SM	NSM	SM	NSM
Y15 × RC2-10	Sc × Su direct spore-to-spore	0	23	10	28
Y21 × NBRC1948	Sc × Sbay direct spore-to-spore	13	33	39	55

## Data Availability

Data sharing not applicable.

## Supplementary Materials

The following are available online at https://www.mdpi.com/2076-2607/9/3/514/s1. Figure S1: Confirmation of hybridization by (A) ITS1 PCR-RFLP with endonuclease *Hae*III and (B) amplification of *FSY1* and *MEX67* genes using species-specific primers; Figure S2: Correlations plots between expected and actual values of ethanol concentrations (A) and yeast dry matter (B); Table S1: Hybrid sporulation assay. Table S2: Wort fermentation parameters in laboratory scale trials (15 °P, 20 °C).
