# Supplementary material for: Hybridization of Saccharomyces cerevisiae Sourdough Strains with Cryotolerant Saccharomyces bayanus NBRC1948 as a Strategy to Increase Diversity of Strains Available for Lager Beer Fermentation
Source: Microorganisms. 2021 Mar 2;9(3):514. doi: 10.3390/microorganisms9030514 (PMC8000887; doi:10.3390/microorganisms9030514)
Supplement: Supplementary file 1 [file microorganisms-09-00514-s001.pdf]

microorganisms

MDPI

Article

Hybridization of *Saccharomyces cerevisiae* sourdough strains with cryotolerant *Saccharomyces bayanus* NBRC1948 as a strategy to increase diversity of strains available for lager beer fermentation

Martina Catallo, Fabrizio Iattici , Cinzia L. Randazzo, Cinzia Caggia, Kristoffer Krogerus, Frederico Magalhães, Brian Gibson and Lisa Solieri *

1. Materials and Methods

1.1 Hybrid validation

For molecular validation of hybrid candidates, yeast DNA was extracted from single colonies after at least two rounds of streaking with the lithium acetate-SDS method [1] and submitted to PCR-RFLP analysis of ITS1 spacer with *Hae*III enzyme (Thermo Fisher Scientific, Waltham, MA, USA) [2] and PCR amplifications of *FSY1* and *MEX67* genes using species-specific primers [3]. All PCR reactions were carried out with a T100 Thermal Cycler (BioRad, Hercules, CA, USA) in 20 µL of final volume containing 1 µL of colony DNA as template, 0.4 µM of each primer, 200 µM each dNTP, and 0.5 U DreamTaq DNA polymerase (Thermo Fisher Scientific, Waltham, MA, USA) according to the manufacturer's instructions. GeneRuler 100 bp Plus DNA (Thermo Scientific, Waltman, MA, USA) was as molecular weight marker.

1.2 Sporulation test of hybrids

Yeast hybrids were sub-cultured in YPDA medium (1% w/v yeast extract, 1% w/v peptone, 2% w/v dextrose, 2% w/v agar) at 28 °C for 24 h, transferred to sporulation medium (ACM; 0.5% w/v sodium acetate, 2% w/v agarose; pH 6.5) and incubated at 28 °C for a period of 14 days. Asci formation was microscopically checked after 3, 7, and 14 days and scored according to Kurtzman et al. [4].

1.2 Wort fermentations

Micro-scale trials of hybrids and their parents were carried out according to Catallo et al. [5]. Fermentations were performed in duplicate with 100 mL of 15 °Plato (°P) all-malt wort (96.36 g/L maltose and 40.18 g/L maltotriose) in 250 mL Erlenmeyer flasks, without agitation. Airlocks containing 2 mL of 85% glycerol were used to seal the flasks. Fermentation progress was monitored by measuring periodically mass loss from the fermenters due to CO_2 release with an analytical balance. A 'neutral' fermentation temperature of 20 °C was chosen to support growth of all strains. Final measurements and samples were taken after 14 days. Ethanol concentration and pH values were determined from the centrifuged and degassed fermentation samples using an Anton Paar Density Meter DMA 5000 M with Alcolyzer Beer ME and pH ME modules (Anton Paar GmbH, Austria). After washing with deionized H_2O, each yeast pellet was transferred to a pre-weighed porcelain crucible, dried overnight at 105 °C and weighed to determine the dry mass content. Viability percentage was determined in a NucleoCounter® YC-100™ as previously reported [3] and calculated as follows:

$$\% \text{ viability} = [(\text{total cells} - \text{dead cells}) / \text{total cells}] \times 100$$

Fermentation curves were modelled based on the weight loss trend over time using the 'grofit'-package for R [6]. Maximum rate of fermentation µ (h^{-1}) and maximum fermentation efficiency A (%

w/v CO_2 released at the end of the fermentation) were determined using the spline-fitting method in 'grofit'. Expected ethanol concentration (% w/v), yeast dry mass (% w/v) and fermentable sugar consumed (% w/v) were calculated using A values based on Balling equation [7]:

$$2.0665 \text{ g extract} = 1.0000 \text{ g alcohol} + 0.9565 \text{ g } CO_2 + 0.11 \text{ g yeast dry matter}$$

Theoretical values of ethanol concentrations (% w/v) were calculated from maximum fermentation efficiency values (A) assuming that:

$$1\ mole\ CO_2\ released\ = 1\ mole\ C_2H_5OH\ produced$$

Ethanol concentration and pH values were determined from the centrifuged and degassed fermentation samples using an Anton Paar Density Meter DMA 5000 M with Alcolyzer Beer ME and pH ME modules (Anton Paar GmbH, Graz, Austria). After washing with deionized H_2O, each yeast pellet was transferred to a pre-weighed porcelain crucible, dried overnight at 105 °C and weighed to determine the dry mass content.

2. Results

2.1 Molecular validation of hybrids

Two independent molecular assays targeting rDNA ITS1 and the protein-encoding genes *FSY1* and *MEX67* were used for validated the hybrid status of 46 candidates out of 190 attempted crosses. As showed in Supplementary **Figure S1**, hybrids contained ITS copies from both the parents and were positive to both the *S. cerevisiae* and *S. eubayanus*-specific PCRs.

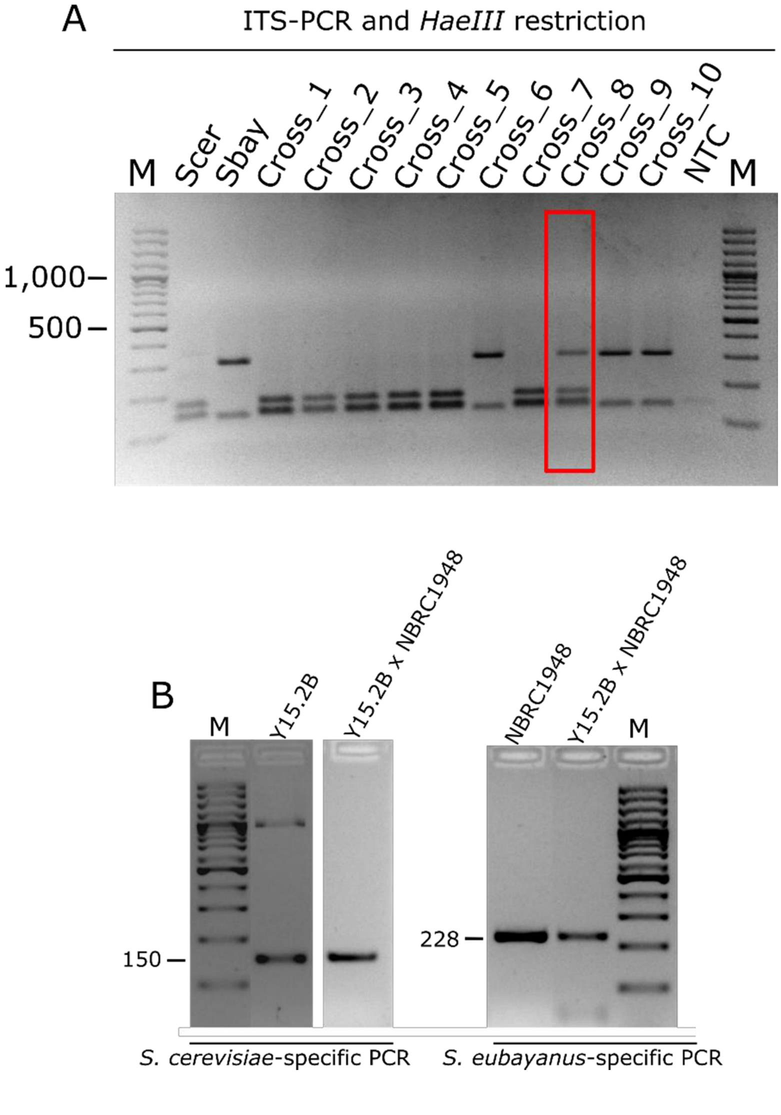

Figure S1. Confirmation of hybridization by (A) ITS1 PCR-RFLP with endonuclease *Hae*III and (B) amplification of *FSY1* and *MEX67* genes using species-specific primers. Abbreviations: M, molecular weight marker; Scer, *S. cerevisiae*; Sbay, *S. bayanus* NBRC1948; NTC, negative control.

2.2 Sporulation test

Results of sporulation tests were reported in **Table S1**. All Scer x Sbay and Scer × Scar hybrids were able to sporulate, while Scer × Su hybrids were more recalcitrant to undergo meiosis.

Table S1. Hybrid sporulation assay. Strains sporulating on ACM medium after 3 and 7 days were scored as + and w (weak), respectively, while no sporulating strains were scored as -.

Crosses	Hybrids	Sporulation (days)		
		3	7	14
Sc × Su[1]	Y23.7A×RC2-10.4A	-	-	-
	Y23.10B × RC2-10.7B	-	-	-
	Y23.10D × RC2-10.7D	+	+	+
Sc x Sbay	Y15.2B × NBRC 1948	-	+	+
	Y19.11B × NBRC1948	+	+	+
	Y19.12B × NBRC1948	+	+	+
	Y19.12C × NBRC1948	+	+	+
	Y19.13C × NBRC1948	+	+	+
	Y21.7B × NBRC1948	+	+	+
	Y21.9C × NBRC1948	+	+	+
	Y21.10A × NBRC1948	-	w	w
Sc x Scar	Y19.8A × CBS 8841.2A	-	w	w
	Y19.8C × CBS 8841.2C	+	+	+
	Y19.5A.1B × CBS 8841.4B	+	+	+

[1] Abbreviations: Sc, *S. cerevisiae*; Su, *S. uvarum*; Sbay, *S. eubayanus* x *S. uvarum*; Scar, *S. cariocanus*.

2.3 Wort fermentations

Three Sc x Sbay and 2 Sc x Su hybrids were screened for their ability to ferment 15 °P wort at 20°C. Ethanol production and viability were higher for Sc × Sbay hybrids compared to Sc × Su hybrids. Hybrid Y15.2B × NBRC1948

Table S2. Wort fermentation parameters in laboratory scale trials (15 °P, 20 °C). Different superscript letters indicate significant differences ($p < 0.05$) in the same column, as determined by one-way ANOVA with post-hoc Tukey HSD test. Superscript letters were attributed as follows: the highest value was marked as 'a', the next value that is significantly different can be 'b', and so on. Abbreviations: Sc, *S. cerevisiae*; Sbay, *S. eubayanus* x *S. uvarum*; Su, *S. uvarum*; H, hybrid.

Species	Strains	Ethanol (% v/v)	Ethanol yield[1]	pH	Viability (%)	Dry mass (%)
Su	RC2-10	5.81 ± 0.03^{e}	69.13 ± 0.34^{f}	4.4 ± 0.00^{b}	43.3 ± 5.01^{b}	0.164 ± 0.011^{a}
H	Y23 × RC2-10	6.6 ± 0.01^{c}	77.78 ± 0.17^{d}	4.5 ± 0.02^{a}	85.0 ± 1.97^{a}	0.169 ± 0.001^{a}
Sc	Y23	7.2 ± 0.01^{a}	85.31 ± 0.08^{b}	4.5 ± 0.01^{a}	58.1 ± 14.65^{b}	0.156 ± 0.006^{a}
Sc	Y15.2B	7.2 ± 0.09^{a}	85.49 ± 0.17^{ab}	4.4 ± 0.01^{b}	72.9 ± 0.78^{a}	0.07 ± 0.02^{c}
Sc	Y15.2B × NBRC1948	7.2 ± 0.02^{a}	84.84 ± 0.42^{b}	4.4 ± 0.01^{b}	72.6 ± 5.62^{a}	0.07 ± 0.01^{c}
Sbay	NBRC1948	7.1 ± 0.01^{b}	84.72 ± 0.08^{bc}	4.5 ± 0.01^{a}	70.8 ± 11.59^{ab}	0.09 ± 0.01^{bc}
Sc	Y19	7.3 ± 0.03^{a}	86.67 ± 0.34^{a}	4.5 ± 0.01a	73.8 ± 11.35^{ab}	0.155 ± 0.005^{ab}
H	Y19 × NBRC1948	7.05 ± 0.01^{b}	83.53 ± 0.08^{c}	4.4 ± 0.05^{b}	77.1 ± 3.36^{ab}	0.179 ± 0.008^{ab}
Sc	Y21	7.2 ± 0.04^{a}	84.90 ± 0.50^{b}	4.3 ± 0.02^{c}	80.7 ± 1.86^{a}	0.165 ± 0.010^{ab}
H	Y21 × NBRC1948	7.0 ± 0.03^{b}	82.52 ± 0.34^{c}	4.3 ± 0.03^{c}	79.8 ± 1.07^{a}	0.208 ± 0.001^{a}
Sc	3002	6.00 ± 0.01^{d}	71.08 ± 0.08^{e}	4.3 ± 0.03^{c}	76.0 ± 19.0^{ab}	0.11 ± 0.05^{bc}
H	LS3	6.02 ± 0.04^{d}	71.32 ± 0.42^{e}	4.5 ± 0.01^{a}	44.6 ± 8.22^{b}	0.13 ± 0.03^{b}
Su	7877	6.03 ± 0.01^{d}	71.44 ± 0.08^{e}	4.4 ± 0.01^{b}	34.1 ± 4.19^{b}	0.06 ± 0.04^{c}

[1] Ethanol yield was calculated as percentage of theoretical ethanol concentration considering maltose and maltotriose concentrations of 96.36 and 40.18 g/L, respectively.

Maximum fermentation efficiency values (A) resulted from Spline-based fitting of weight loss curves were used to calculate expected fermentable extract consumed (% w/v), expected ethanol production (% w/v) and expected yeast dry mass (% w/v), according to Balling equation. In Supplementary **Figure S2** actual values of ethanol and yeast dry matter were correlated to those calculated from Balling equation.

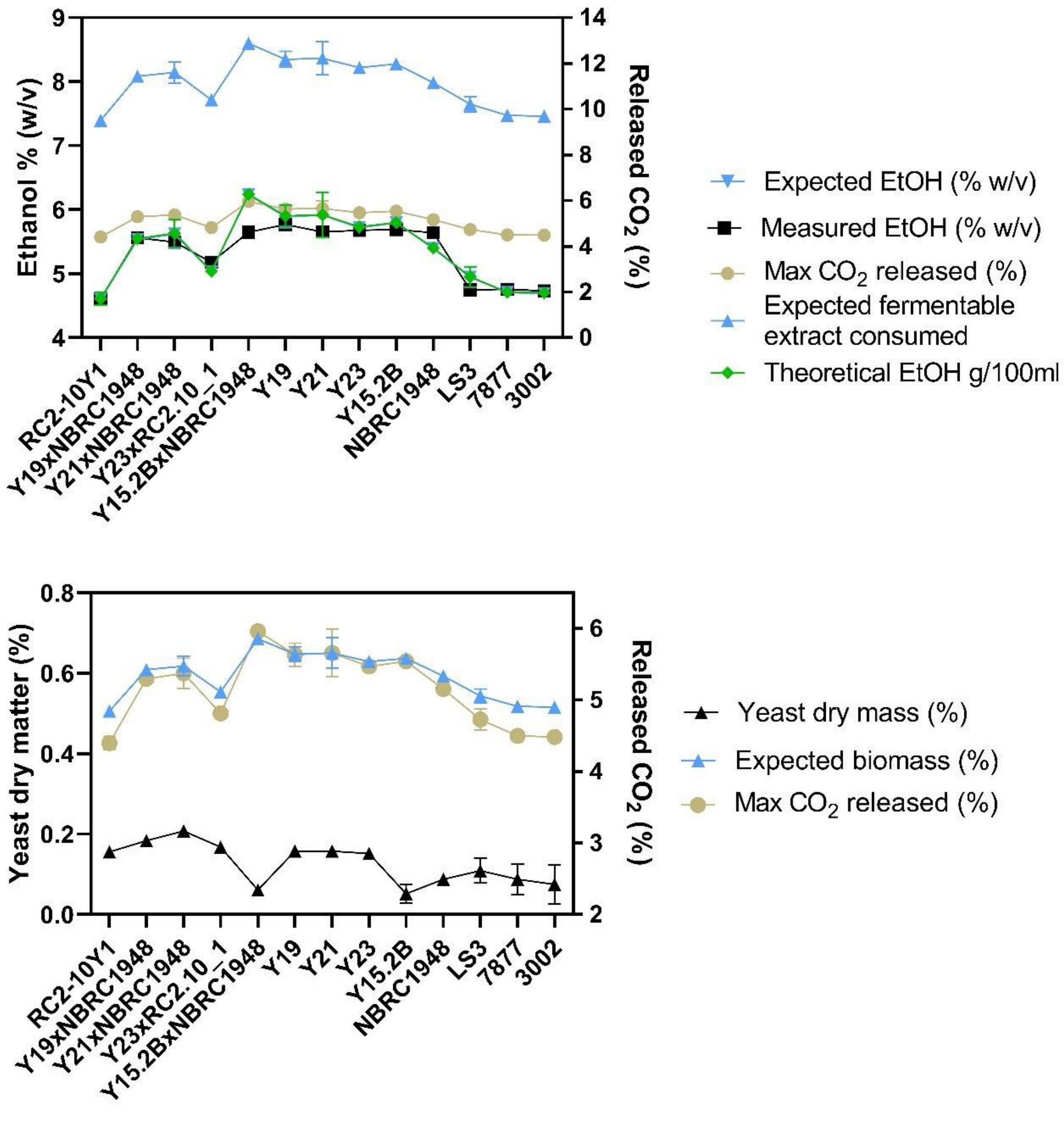

Figure S2. Correlations plots between expected and actual values of ethanol concentrations (A) and yeast dry matter (B). Stoichiometric estimations of ethanol, fermentable extract consumed and yeast dry matter were predicted by Balling equation considering A values from spline-fitting, while actual values were measured at the end of laboratory-scale fermentation trials in wort 15 °P (20 °C). Theoretical ethanol concentration was calculated assuming the equation of Lavoisier and Gay-Lussac (1 mole CO_2 released corresponds to 1 mole glucose). Abbreviations: EtOH, ethanol.

References

1. Lõoke, M.; Kristjuhan, K.; Kristjuhan, A. Extraction of genomic DNA from yeasts for PCR-based applications. *Biotech.* **2011**, *50*, 325–328.
2. Solieri, L.; Verspohl, A.; Bonciani, T.; Caggia, C.; Giudici P. Fast method for identifying inter- and intra-species *Saccharomyces* hybrids in extensive genetic improvement programs based on yeast breeding. *J. Appl. Microbiol.* **2015**, *119*, 149–161.
3. Pengelly, R.J.; Wheals, A.E. Rapid identification of *Saccharomyces eubayanus* and its hybrids. *FEMS Yeast Res.* **2013**, *13*, 156–161.

4. Kurtzman, C.P.; Fell J.W.; Boekhout, T.; Robert, R. 2011. Methods for Isolation, Phenotypic Characterization and Maintenance of Yeasts. In *The Yeasts: A Taxonomic Study*, 5th ed.; Kurtzman, C. P., Fell, W. F., Boekhout, T., Eds; Elsevier Elsevier, Amsterdam, The Netherlands, **2011**, *1*,87–110.
5. Catallo, M.; Nikulin, J.; Johansson, L.; Krogerus, K.; Laitinen, M.; Magalhães, F.; Piironen, M.; Mikkelson, A.; Randazzo, C.L.; Solieri, L.; Gibson, B. Sourdough derived strains of *Saccharomyces cerevisiae* and their potential for farmhouse ale brewing. *J. Inst. Brew.* **2020**. doi.org/10.1002/jib.608.
6. Kahm, M.; Hasenbrink, G.; Lichtenberg-frate, H.; Ludwig, J.; Kschischo, M. Grofit: fitting biological growth curves. *J. Stat. Softw.* **2010**, *33*:1–21.
7. Esslinger, H.M. Handbook of Brewing: Processes, Technology, Markets; John Wiley & Sons: Hoboken, NJ, USA.
